# Cannabis use among primary care patients with depression and anxiety disorders: cross-sectional analysis in a large health system in Los Angeles, CA

**DOI:** 10.1186/s42238-025-00354-z

**Published:** 2025-12-29

**Authors:** Julia Koerber, Marjan Javanbakht, Naira Setrakian, Un Young Rebecca Chung, Whitney N. Akabike, Lawrence Dardick, Ziva D. Cooper, Lillian Gelberg

**Affiliations:** 1 UCLA Fielding School of Public Health, Department of Epidemiology, CHS 46-082, Box 951772, Los Angeles, CA 90095-1772 USA; 2 UCLA David Geffen School of Medicine, Department of Medicine, Statistics Core, Los Angeles, CA USA; 3 Department of Family Medicine, UCLA David Geffen School of Medicine, Los Angeles, CA USA; 4 UCLA Jane and Terry Semel Institute for Neuroscience and Human Behavior, UCLA Center for Cannabis and Cannabinoids, Los Angeles, CA USA; 5 UCLA David Geffen School of Medicine, Department of Psychiatry and Biobehavioral Sciences, University of California, Los Angeles, CA USA; 6 UCLA David Geffen School of Medicine, Department of Anesthesiology and Perioperative Medicine, University of California, Los Angeles, CA USA; 7 UCLA Fielding School of Public Health, Department of Health Policy and Management, Los Angeles, CA USA

**Keywords:** Cannabis use, Primary care, Mental health care, Depression, Anxiety

## Abstract

**Background:**

Depression, anxiety, and cannabis use are growing, interconnected primary care concerns, but remain understudied due few health systems conducting systematic cannabis use screening. This study examines cannabis use and risk of cannabis use disorder (CUD) among primary care patients, comparing outcomes by depression and anxiety diagnoses and psychotropic prescriptions.

**Methods:**

We assessed past three-month cannabis use, reasons for use, and risk of CUD among 170,032 adult primary care patients at a large health system in Los Angeles, CA under a routine screening protocol using the validated, self-administered ASSIST survey. This survey was embedded in the electronic health record (EHR), where data on ICD-10 diagnostic codes for depressive and anxiety disorders, psychotropic prescriptions, and demographics were collected. Logistic regression analysis assessed the association of depression and anxiety diagnoses on risk of CUD.

**Results:**

Median age was 48 years (IQR 35–61), 57.8% were female, and 17.5% reported cannabis use. 24.0% had anxiety diagnoses and 9.0% had depression diagnoses. Cannabis use was higher among patients diagnosed with depression or anxiety than other patients (21.7%-27.4% vs. 15.5%, *p* < 0.001). Most patients who reported cannabis use and had depression or anxiety diagnoses reported using cannabis to manage emotional symptoms (62.5%-71.3%); notably, 47.3% of these patients had current antidepressant or anxiolytic prescriptions. Moderate-to-high risk of CUD was elevated among patients who reported cannabis use diagnosed with depression (9.8%), anxiety (8.0%), and particularly depression and anxiety (12.9%) (other patients = 4.9%; *p* < 0.001). After adjusting for age, sex, race and ethnicity, sexuality (LGB + versus heterosexual), employment, and Charlson Comorbidity Index, depression and anxiety diagnoses were associated with elevated risk of CUD [aOR 1.99 (95% CI 1.78–2.23); aOR 1.6 (95% CI 1.51–1.69), respectively], with the highest association among patients with both diagnoses [aOR 2.58 (95% CI 2.34–2.83)].

**Conclusions:**

Clinical depression and anxiety diagnoses were associated with elevated cannabis use prevalence and risk of CUD. Many primary care patients reported using cannabis to manage mental health-related symptoms, even when prescribed psychotropic medications. These findings highlight the need for providers to assess and address cannabis use and potential CUD among primary care patients, especially those diagnosed with depression or anxiety.

**Trial registration:**

Clinical trial number: not applicable.

## Introduction

Cannabis use is increasing in the United States, and adults with anxiety or depressive disorders are more likely to use cannabis than other adults (Weinberger et al. 2020; Gorfinkel et al. 2020; Pacek et al. 2020). In the 2021 National Survey on Drug Use and Health household survey, 18.7% of people 12 and older reported past-year cannabis use, increasing to 33.8% among adults with mental illness. (Center for Behavioral Health Statistics and Quality 2021) In the context of expanding access to cannabis and increasing prevalence of depression and anxiety disorders, (Haomiao et al. 2021) assessing cannabis use among patients with anxiety and depression is important for managing mental health in primary care.

Delta-9-tetrahydrocannabinol (THC), the primary psychoactive component of the cannabis plant, has wide-ranging biological effects that can cause various acute effects, including relaxation, euphoria, and pain reduction (Borgelt et al. 2013). Concordantly, in national surveys and surveys of medical cannabis dispensary customers, people who use cannabis commonly report cannabis use to manage anxiety and depressive symptoms as well as physical symptoms related to mental health, including pain and sleep difficulties (Kosiba et al. 2019; Leung et al. 2022; Turna et al. 2019). However, the findings on the potential impact of cannabis on anxiety and depression are mixed. Some observational research shows no impact of cannabis on depression while some suggests that cannabis exacerbates outcomes, and limited clinical trial data have also not indicated that THC improves depression outcomes (Stanciu et al. 2021; Polkosnik et al. 2021; Sorkhou et al. 2024). While there is more promising data of cannabis improving anxiety, the available studies are heterogeneous and limited (Beletsky et al. 2024, Roberts et al. 2025). Altogether, this raises the concern that patients using cannabis to manage mental health symptoms may be masking or potentially exacerbating underlying depression or anxiety.

Understanding whether primary care patients with anxiety and depression use cannabis to manage their mental health is important: it may reveal gaps in care, inform best practices in psychiatric medication prescription, and guide patient interactions with their primary care provider. Despite these potential clinical implications, research on reasons why primary care patients with depression and anxiety use cannabis is limited, as is information on concurrent cannabis use among patients with prescriptions for anti-depressant and anxiety medications. We examined antidepressant and/or anxiolytic use to quantify the prevalence of co-use, flag potential under-treatment suggested by adjunctive cannabis use, and identify polypharmacy risks so clinicians can counsel patients and optimize care.

Our study analyzed diagnosis and prescription records from the EHR alongside results from a patient self-administered cannabis use screening questionnaire electronically delivered to every adult primary care patient in a large healthcare system in California, which legalized medical cannabis use in 1996 and recreational adult use in 2016 (National Conference of State Legislatures 2022). This study examines the prevalence of cannabis use, risk for CUD, and reasons for cannabis use to assess if patients with depression and anxiety diagnoses should be considered a priority population for CUD screening and cannabis use counseling in primary care. We hypothesized that the prevalence of cannabis use and risk of CUD would be higher among patients diagnosed with depression or anxiety than among patients with neither diagnosis, but would be lower among patients diagnosed with depression or anxiety who were prescribed psychotropic medication.

## Methods

### Study setting

We utilized data from a large academic health system in Los Angeles, encompassing four hospitals and over 200 affiliated primary care clinics (UCLA Health 2021). Beginning in January 2021, all adult patients (≥ 18 years) scheduled for either a new patient or annual primary care visit were asked to complete a cannabis screening questionnaire. The details of the cannabis screener are described elsewhere (Gelberg et al. 2024). The screener is sent to every patient through the online patient portal (“MyChart”), and has an estimated response rate of 87.8% among patients who access MyChart (Gelberg et al. 2024). This analysis includes EHR data including sociodemographic factors, clinical characteristics, and cannabis screening from 170,032 unique patients’ annual primary care visits between January 2021 to June 2023.

### Ethics and consent

This study is a secondary analysis of deidentified EHR data. The Institutional Review Board at University of California, Los Angeles reviewed and approved this project, and waived informed consent in accordance with the US Department of Health and Human Services Office for Human Research Protections Common Rule.

### Measures

#### Cannabis use outcome variables

The TCQ was adapted from the National Institute of Drug Abuse’s modification (NIDA 2012) of the validated World Health Organization’s Alcohol Substance Involvement Screening Test (ASSIST) version 3.0. (Humeniuk et al. 2008; WHO Assist Working Group 2002) Questions related to cannabis use included past three-month frequency of 1) use, 2) craving, 3) negative consequences due to use, 4) hindered productivity due to use, 5) concern about use from loved ones and 6) attempts to decrease use. Response options for frequency of use in the past 3 months included daily, weekly, monthly, once or twice, and never use. Further, each item in the ASSIST was scored using the standard scoring algorithm, with increasing scores suggesting higher risk of CUD. Based on prior research and adaptation for the primary care setting, (Gelberg et al. 2024) scores were categorized into levels of no or low risk (scores 0–7), moderate risk (Leung et al. 2022; Turna et al. 2019; Stanciu et al. 2021; Polkosnik et al. 2021; Sorkhou et al. 2024; Beletsky et al. 2024, Roberts et al. 2025, National Conference of State Legislatures 2022, UCLA Health 2021, NIDA 2012, World Health Organization 1992; Gelberg et al. 2024; Humeniuk et al. 2008; WHO Assist Working Group 2002; Centers for Disease Control and Prevention (CDC) (n,d).; Lapham et al. 2022, Lapham et al. 2017; Schauer et al. 2016; Charlson et al. 1987), and high risk (> 26), (Humeniuk et al. 2008; WHO Assist Working Group 2002). This analysis compared patients at moderate-to-high risk for CUD (ASSIST score ≥ 8) to patients with no/low risk of CUD (ASSIST score 0–7). Additionally, the cannabis questionnaire also assessed if patients used cannabis in the past 3 months to manage mental health-related symptoms, including stress, worry or anxiety, depression or sadness, sleep, and pain. (Centers for Disease Control and Prevention (CDC) (n.d.); Lapham et al. 2022; Schauer et al. 2016).

#### Clinical factors

*Clinical diagnosis of depressive or anxiety disorders* was based on the ICD-10 (International Classification of Disease-10) codes available in the problem or current diagnosis lists listed in the EHR during the visit when patients completed TCQ screening. Anxiety and depressive diagnoses were based on ICD-10 stem codes F32, F33, F40, and F41 (World Health Organization 1992). *Relevant prescription medications* were identified in the medication list from the same visit, and included benzodiazepines, selective serotonin reuptake inhibitors (SSRIs), serotonin-norepinephrine reuptake inhibitors (SNRIs), or other antidepressants (amitriptyline, nortriptyline, trazodone, mirtazapine, and bupropion). We used these prescription data to create a binary summary variable to reflect which medications a patient was prescribed at the time of their primary care visit. *Comorbidity status* was measured using the Charlson Co-morbidity Index (CCI) with scores based on ICD-10 codes for relevant conditions (Charlson et al. 1987). The model controlled for CCI to reduce confounding from other health conditions potentially contributing to the association between depression or anxiety diagnoses and risk of CUD.

#### Patient demographics

Demographic information was collected from the EHR, including age, sex, race and ethnicity, sexual identity, and employment status. Race and ethnicity data were self-reported and categorized as Asian or Pacific Islander, African American or Black, Hispanic or Latino, another race or ethnicity (including individuals who identified as Middle Eastern or North African, American Indian or Alaskan Native, multiracial, or as another race or ethnicity), or white. Sexual identity was categorized as lesbian, gay, bisexual, or other non-heterosexual identities (LGB +) or heterosexual. Employment was categorized as employed (which includes full and part time workers and students, retired and disabled individuals, and active military) or as unemployed/not working.

### Analysis

Descriptive statistics examined the prevalence of past three-month cannabis use, prevalence of being at moderate-to-high risk for cannabis use disorder, and reasons for cannabis use comparing patients with and without a diagnosis of depression and/or anxiety. Differences were assessed using ANOVA, Kruskal–Wallis tests, chi-square tests or Fisher’s exact tests as appropriate. Additional descriptive statistics examined reasons for cannabis use, examining differences across patients prescribed or not prescribed antidepressant or anxiolytic medications. Multivariable logistic regression examined the association between anxiety and/or depression diagnoses and moderate-to-high risk of CUD. The models adjusted for age, sex, race and ethnicity, sexuality, employment status, and comorbidity status (using CCI). The analyses were conducted in R, version 4.3.1, using the packages dplyr, tidyr, data.table, janitor, flextable, and gtsummary.

## Results

### Patient demographics and mental health characteristics

Among the 170,032 patients screened between January 2021 and June 2023, the median age was 48 years (IQR 35–61), more were female (57.8%), most were employed (88.3%), and 14.5% identified as Asian or Pacific Islander, 4.0% as African American or Black, 12.5% as Hispanic or Latino, 28.8% as white, and 32.0% with another racial or ethnic background (Table 1). Almost one-quarter (24.0%) of patients had an anxiety diagnosis and 9.0% had a depression diagnosis. Examining diagnoses jointly, 17.9% of patients had an anxiety diagnosis only, 3.0% had depressive diagnosis only, and 6.1% had both diagnoses. Compared to patients not diagnosed with depression or anxiety, those with both diagnoses were younger (median age 43 vs. 49; *p* < 0.001), more likely to be female (70.5% vs. 54.3%; *p* < 0.001), and had more comorbidities (CCI score 1.3 vs. 0.7; *p* < 0.001). Among patients with depressive diagnoses, 46.2% were prescribed at least one antidepressant or anxiolytic medication, as were 39.2% of patients with anxiety diagnoses, and 61.8% of patients with both diagnoses.

**Table 1:** Characteristics of primary care patients screened for cannabis use, Jan. 2021-June 2023

	**Total (*n* = 170,032) n (%)**	**Depression Diagnosis (*n* = 5,020) n (%)**	**Anxiety Diagnosis (*n* = 30,405) n (%)**	**Depression and Anxiety Diagnoses (*n* = 10,320) n (%)**	**Neither Diagnosis (*n* = 124,287) n (%)**
Age, years, median (min–max)**	48 (18–103)	51 (18–99)	45 (18–99)	43 (18–97)	49 (18–103)
18–29	23290 (13.7)	689 (13.7)	4890 (16.1)	2052 (19.9)	15659 (12.6)
30–39	34959 (20.6)	898 (17.9)	7014 (23.1)	2387 (23.1)	24660 (19.8)
40–49	32533 (19.1)	783 (15.6)	5863 (19.3)	1852 (18.0)	24035 (19.3)
50–59	31232 (18.4)	882 (17.6)	5190 (17.1)	1574 (15.3)	23586 (19.0)
60 +	48018 (28.2)	1768 (35.2)	7448 (24.5)	2455 (23.8)	36347 (29.2)
Sex**					
Male	71599 (42.1)	1764 (35.1)	10044 (33.1)	3023 (29.3)	56768 (45.7)
Female	98286 (57.8)	3248 (64.7)	20334 (66.9)	7280 (70.5)	67424 (54.3)
Sexuality**					
LGB +	11344 (6.7)	583 (11.6)	2548 (8.4)	1520 (14.7)	6693 (5.4)
Heterosexual	142471 (83.8)	3952 (78.7)	25242 (83.0)	7866 (76.2)	105411 (84.8)
Race and ethnicity**					
Asian or Pacific Islander	24575 (14.5)	482 (9.6)	2854 (9.4)	903 (8.8)	20336 (16.4)
African American or Black	6806 (4.0)	204 (4.1)	1060 (3.5)	393 (3.8)	5149 (4.1)
Hispanic or Latino	21246 (12.5)	583 (11.6)	4024 (13.2)	1540 (14.9)	15099 (12.2)
Another race or ethnicity	54469 (32.0)	1795 (35.8)	10657 (35.1)	3733 (36.2)	38284 (30.8)
White	48881 (28.8)	1578 (31.4)	9445 (31.1)	3018 (29.2)	34840 (28.0)
Unemployment**					
Yes	19881 (11.7)	643 (12.8)	4033 (13.3)	1421 (13.8)	13784 (11.1)
No	150151 (88.3)	4377 (87.2)	26372 (86.7)	8899 (86.2)	110503 (88.9)
Prescribed antidepressant and/or anxiolytic medications**					
SSRIs	13,363 (7.9)	1,241 (24.7)	5,689 (18.7)	3,176 (36.0)	2,717 (2.2)
SNRIs	3,145 (1.9)	361 (7.2)	945 (3.1)	961 (9.3)	878 (0.7)
Benzo-diazepines	10,689 (6.3)	302 (6.0)	5,498 (18.1)	1,986 (19.2)	2,903 (2.3)
Other anti-depressants	8,821 (5.2)	951 (18.9)	2,530 (8.3)	2,404 (23.3)	2,936 (2.4)
At least one of the above	28,976 (17.0)	2,320 (46.2)	11,919 (39.2)	6,377 (61.8)	8,360 (6.7)
Charlson Comorbidity Index, mean, (SD) **	0.8 (1.9)	1.2 (2.3)	1 (2.2)	1.3 (2.5)	0.7 (1.7)

*LGB* + Lesbian, gay, bisexual, or non-heterosexual. *SSRI* Selective serotonin reuptake inhibitors. *SNRIs* Serotonin-norepinephrine reuptake inhibitor

Other anti-depressants include amitriptyline, nortriptyline, trazodone, mirtazapine, and bupropion

Percentages may not sum to 100% due to missing data or rounding

^**^ = *p*-value < 0.001

### Prevalence of cannabis use and risk of CUD, by diagnoses

The prevalence of past three-month cannabis use was 17.5% overall and higher among patients with depression (22.6%), anxiety (21.7%), or both diagnoses (27.4%) compared to patients with neither diagnosis (15.5%) (Table 2), *p*-value < 0.001). Likewise, the prevalence of scoring at moderate-to-high risk of CUD among patients who reported cannabis use was highest among those diagnosed with both depression and anxiety (12.9%), followed by those with depression (9.8%), and those with anxiety (8.0%) compared to 4.9% among patients with neither diagnosis (*p* < 0.001).

**Table 2:** Cannabis use among primary care patients by depression and anxiety diagnoses, January 2021-June 2023

	**Neither Diagnosis (*n* = 124,287) n (%)**	**Depression Diagnosis (*n* = 5,020) n (%)**	**Anxiety Diagnosis (*n* = 30,405) n (%)**	**Depression and Anxiety Diagnoses (*n* = 10,320) n (%)**
CUD risk, past 3-months**				
No risk^	10,5042 (84.5)	3,886 (77.4)	23,806 (78.3)	7,490 (72.6)
Low risk	13,155 (10.6)	643 (12.8)	4,183 (13.8)	1,504 (14.6)
Moderate-to-high risk	6,090 (4.9)	491 (9.8)	2,416 (8.0)	1,326 (12.9)
**Symptoms patients reported using cannabis to manage, *among patients who reported any past 3-month cannabis use (CU)***				
**Patients who reported any cannabis use, past 3 months**	**Neither Diagnosis, reported CU (*n* = 19,245) n (%)**	**Depression Diagnosis, reported CU (*n* = 1,134) n (%)**	**Anxiety Diagnosis, reported CU (*n* = 6,599) n (%)**	**Depression and Anxiety Diagnoses, reported CU (*n* = 2,830) n(%)**
**Symptoms managed with cannabis**				
Stress**	8,236 (42.8)	699 (61.6)	3,720 (56.4)	1,832 (64.7)
Worry/anxiety**	5,306 (27.6)	515 (45.4)	3,085 (46.8)	1,583 (55.9)
Depression/sadness**	2,149 (11.2)	365 (32.2)	1,366 (20.7)	1,100 (38.9)
Any emotional symptom**	9,145 (47.5)	755 (66.6)	4,123 (62.5)	2,018 (71.3)
Sleep**	5,413 (28.1)	677 (59.7)	4,018 (60.9)	1,824 (64.5)
Pain, non-specific**	*n* = 19,245	414 (36.5)	2,166 (32.8)	1,159 (41.0)

*CUD* Cannabis Use Disorder. Moderate-high risk of CUD = ASSIST score >= 8

^ = includes patients who reported no past three-month cannabis use

Percentages may not sum to 100% because of missing data or rounding

^**^ = *p*-value < 0.001

### Reasons for cannabis use among patients who reported use, by diagnoses

**Fig. 1:**
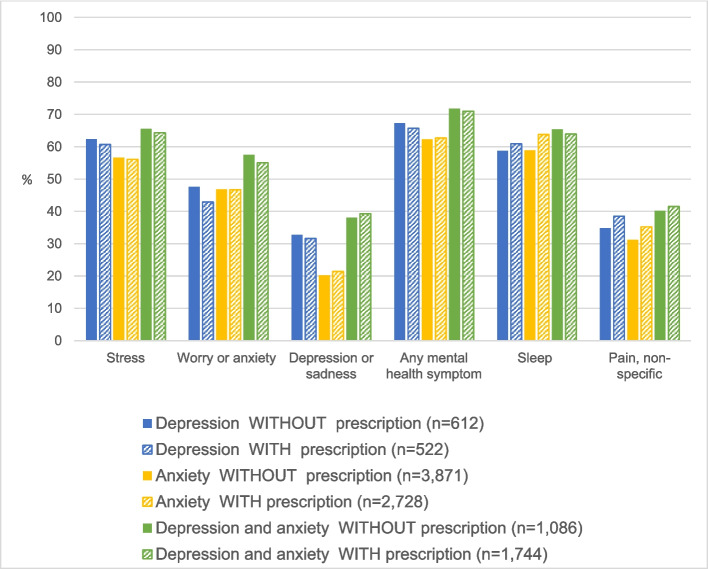
Symptoms managed by cannabis among primary care patients diagnosed with depression or anxiety who reported past three-month cannabis use, by diagnoses and psychotropic prescription status. Anxiety and depressive disorders were identified through ICD-10 stem codes F32, F33, F40, and F41. Medication status was determined by prescription records for benzodiazepines, selective serotonin reuptake inhibitors (SSRIs), serotonin-norepinephrine reuptake inhibitors (SNRIs), and/or other antidepressants (amitriptyline, nortriptyline, trazodone, mirtazapine, and bupropion)

Using cannabis to manage mental health-related symptoms was common among primary care patients who reported cannabis use and elevated among the subset of these patients diagnosed with depression or anxiety (*p*-value < 0.001) (Table 2). The prevalence of using cannabis to manage at least one mental health symptom was highest among patients diagnosed with both depression and anxiety who reported cannabis use, at 71.3%. However, even among patients without depression or anxiety diagnoses, nearly half (47.5%) also reported using cannabis to manage a mental health symptom. Interestingly, the prevalence of cannabis use for management of mental health symptoms was similar among patients diagnosed with depression or anxiety who reported using cannabis regardless of whether these patients were currently prescribed antidepressant or anti-anxiety medications (Fig. 1). For instance, among patients who reported cannabis use, 71.0% of patients diagnosed with both anxiety and depression who were prescribed psychotropic medications reported using cannabis to manage at least one mental health symptom, as did 71.8% of patients with these diagnoses not prescribed psychotropic medication. Figure 1 demonstrates the similar prevalences of reasons for cannabis use among patients diagnosed with depression and/or anxiety who reported cannabis use, across prescription status.

### Factors associated with moderate-to-high risk of cannabis use disorder

**Fig. 2:**
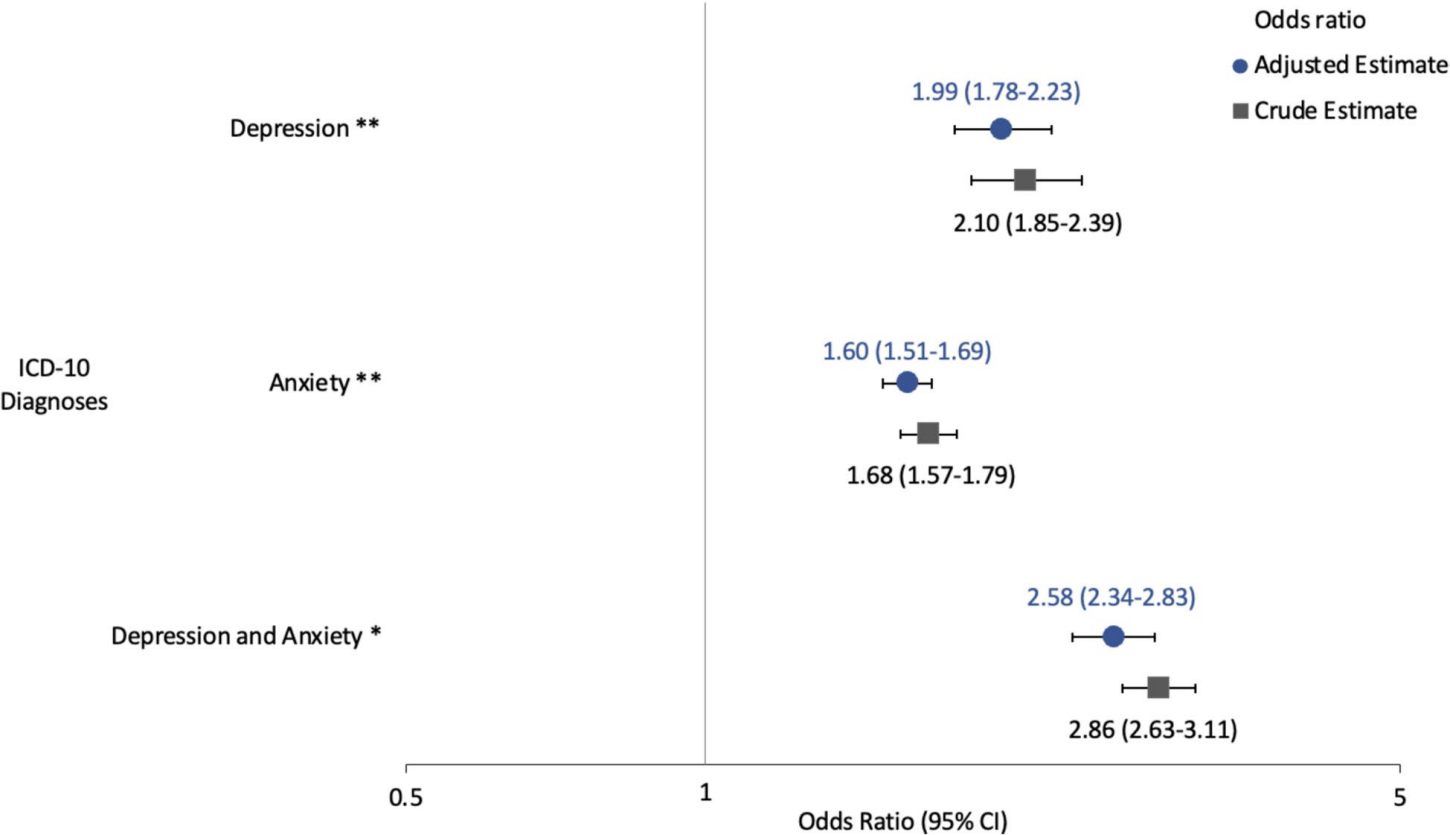
Association of depression and anxiety diagnoses with moderate-high risk of CUD among primary care patients in logistic regression. CUD = Cannabis use disorder. Reference group was patients with neither depression nor anxiety diagnoses. Outcome was moderate-to-high risk versus no/low risk of CUD. Models adjusted for age, sex, race and ethnicity, sexuality, employment, and Charlson Comorbidity Index. X-axis is on a logarithmic scale. ICD-10 = International Classification of Diseases – 10. 95% CI = 95% confidence interval. * = *p*-value for interaction coefficient < 0.01. ** = *p*-value for model coefficient < 0.001

Among patients who reported cannabis use, those diagnosed with depression or anxiety were significantly more likely to be at moderate-to-high risk for cannabis use disorder (CUD) compared to those without these diagnoses (Fig. 2). Based on logistic regression models adjusting for age, sex, race and ethnicity, sexuality, employment status, and CCI score, the odds of being at moderate-to-high risk for CUD were nearly twice for patients with depression (adjusted OR = 1.99, 95% CI 1.78–2.23) and 1.6 times for patients with anxiety (95% CI 1.51–1.69) when compared to patients with neither diagnosis. The risk was even greater for individuals diagnosed with both depression and anxiety, with the adjusted odds of moderate-to-high risk of CUD being 2.6 times that of patients without either diagnosis (95% CI 2.3–2.8).

## Discussion

In one of the first studies to use systematic, self-administered screening for cannabis use among a large primary care clinical population and conducted in a state with legalized medical and recreational cannabis, we found important connections between depression and anxiety diagnoses, prescription treatment, cannabis use, and risk for cannabis use disorder. Cannabis use was common and many patients both with and without diagnosed depression or anxiety reported using cannabis to manage mental health-related symptoms including stress, worry, and sleep disturbances. Among those with diagnosed depression or anxiety, the use of cannabis to manage these symptoms remained high even among patients currently prescribed antidepressant or anxiolytic medications. This suggests that cannabis may be serving as a complementary or alternative approach to conventional treatment for many patients with depression or anxiety. These findings highlight a critical need for routine, informed conversations between patients and providers about cannabis use—particularly among those with depression and anxiety—to ensure satisfactory, safe, and effective care.

Approximately one-fifth of primary care patients with depression or anxiety diagnoses reported cannabis use in the past three months, with one-tenth scoring at moderate-to-high risk of CUD. These prevalences are similar to those identified in prior clinical samples and general population studies assessing depression and anxiety symptoms or diagnoses and cannabis use (Lapham et al. 2017; Kedzior and Laeber 2014; Hunt et al. 2020). Our results also echo prior non-clinical studies that demonstrate that people with depression or anxiety face increased risk of CUD (Onaemo et al. 2021). Our analysis contributes novel findings that compared to other primary care patients, patients with both depression and anxiety diagnoses had the highest prevalence of cannabis use and elevated risk of CUD. Patients with both diagnoses had nearly three times the odds of screening at moderate-to-high risk for CUD compared to those with neither diagnosis, demonstrating a heightened risk that held after adjustment for potential confounders. Altogether, our results suggest that primary care patients diagnosed with depression or anxiety, and particularly patients with comorbid depression and anxiety, may be a priority population for assessing indication of CUD and counseling on cannabis use.

Our study utilized data from a universal cannabis use screener to offer novel insight into the self-reported reasons for cannabis use shared by patients with and without depression and/or anxiety diagnoses. Among cannabis users with these diagnoses, upwards of two-thirds reported using to manage mental health symptoms, which is substantially higher than estimates from a meta-analysis among medical cannabis users (Kosiba et al. 2019). Notably, this pattern did not differ by antidepressant or anxiolytic prescription status. These findings underscore the need for routine discussion of reasons for cannabis use to inform counseling and mental health treatment in primary care. For example, these results may suggest that some patients are using cannabis as a supplement to, or substitute for, prescribed treatments, possibly due to side effects, perceived lack of efficacy, or a desire to reduce medication use altogether (Kvamme et al. 2021; McNabb et al. 2023). The elevated use of cannabis to manage mental health-related symptoms, including among patients already receiving pharmaceutical treatment, may signal undertreated symptoms, gaps in care, or alternative treatment preferences. Importantly, cannabis use alongside psychotropic medications can cause drug-drug interactions, depending on the cannabinoids used and the mode of administration (Vaughn et al. 2021). While this analysis could not account for medication adherence or whether cannabis was medically recommended (Hunt et al. 2020), the findings point to a need for nuanced and proactive clinical engagement about cannabis use that may be better informed by understanding a patient’s reasons for use.

The findings of this study should be interpreted in light of a number of limitations. This was an analysis of adult primary care patients in a large health system in Los Angeles, CA, and results may not apply to other regions or populations. Additionally, the cross-sectional design also means that we cannot distinguish temporality between depression and anxiety diagnoses, symptoms, and cannabis use. It is possible, for example, that cannabis withdrawal may contribute to symptoms of depression and anxiety in some patients, which may contribute to ongoing cannabis use. Since patients may seek mental healthcare from behavioral services externally, some depression and anxiety diagnoses or relevant prescriptions may not be captured in the EHR. We did not have validated mental health symptom screening data, which may have added insight into the patients’ current mental health symptoms. Acknowledging that depression or anxiety may be misclassified or underdiagnosed in a clinical setting, using ICD-10 diagnoses collected from the problem and visit list allowed us to identify clinically relevant cases. Furthermore, using EHR data allowed us to conduct a novel comparison of cannabis use characteristics collected by universal, electronic patient screening by both clinical diagnosis and prescription history, which has yet to be conducted in such a large patient population. The ASSIST is not a diagnostic tool for CUD, so the more conservative cut-off score of 8 points was used to better identify patients who have experienced negative consequences of cannabis use. Patients may not report cannabis use due to stigma and privacy concerns even in a state with legalized access (McNeely et al. 2018). Finally, the presence of a prescription for antidepressant or anxiolytic medication does not necessarily mean that the patient has filled the prescription, is taking the medication, or is adhering to the regimen as prescribed. Despite these limitations, the cannabis screening data provides a more wholistic picture of cannabis use than is available in most large healthcare systems, offering insight into the overlap of cannabis use for mental health symptom management, depression and anxiety diagnoses, and relevant prescription records.

## Conclusions

One-quarter of patients with depression or anxiety diagnoses reported past three-month cannabis use and faced an elevated risk of CUD compared to other patients. Patients self-managing mental health-related symptoms with cannabis was common and was higher among patients with depression and anxiety diagnoses, including those prescribed antidepressant or anxiolytic medications. As such, integrating routine cannabis use screening and structured discussions into primary care, especially for patients diagnosed with depression or anxiety, is essential. These conversations should discuss reasons for use, satisfaction with current medications, and education about potential risks and safer use practices. Doing so can support more holistic, patient-centered care and help address both mental health needs and cannabis-related risks.

## Data Availability

The data used in this study are not publicly due to ensure patient privacy, but may be made available upon reasonable request.

## Abbreviations

CUD: Cannabis use disorder
THC: Delta-9-tetrahydrocannabinol
EHR: Electronic health record
ASSIST: Alcohol Substance Involvement Screening Test
CBD: Cannabidiol
ICD-10: International Classification of Disease-10
SSRI: Selective serotonin reuptake inhibitors
SNRI: Serotonin-norepinephrine reuptake inhibitors
CCI: Charlson Co-morbidity Index
LGB+: Lesbian, gay, bisexual, or other non-heterosexual identities
